# Social distancing and choral singing during the Covid-19 pandemic: challenges and vocal symptoms of chorists

**DOI:** 10.1590/2317-1782/20232021175en

**Published:** 2023-10-23

**Authors:** Diego Henrique da Cruz Martinho, Ana Carolina Constantini, Elisabeth Amin, Mara Suzana Behlau

**Affiliations:** 1 Programa de Saúde Interdisciplinaridade e Reabilitação, Universidade Estadual de Campinas - UNICAMP - Campinas (SP), Brasil.; 2 Departamento de Desenvolvimento Humano, Faculdade de Ciências Médicas, Universidade Estadual de Campinas - UNICAMP - Campinas (SP), Brasil.; 3 Centro de Estudos da Voz - CEV - São Paulo (SP), Brasil.

**Keywords:** Voice, Singing, Coronavirus Infections, Pandemics, Education Distance

## Abstract

**Purpose:**

To analyze the perception of choristers who perform online rehearsals about symptoms, vocal disadvantages, adaptations in the singing routine and difficulties in rehearsals during social distancing due to the COVID-19 pandemic.

**Method:**

Application of an online questionnaire investigating perceptions about the voice and singing routine and rehearsal of 141 choristers who did not have COVID-19 disease and continued in remote activity until April 2021. Participants were divided in two groups according to the age: G1 (18 to 54 years old) and G2 (55 years old or more). The data underwent descriptive and inferential analysis, considering a significance level of 5%.

**Results:**

During distancing, both groups reported a decrease in singing time and felt more difficult to sing alone. Choir singers reported fewer throat infections during this period. Choir singers from G1 noticed a huskier voice, reported difficulty concentrating via videoconferencing, felt nervous to singing alone in virtual rehearsals and stressed recording the same song several times. G2 choristers reported more physical vocal symptoms such as: tired voice, varying throughout the day, and a weaker and more breathy voice.

**Conclusion:**

In social distancing, the choristers found it more difficult to sing by videoconference and had reduced singing practice time. Younger choir singers reported more emotional symptoms and older choir singers reported more vocal symptoms.

## INTRODUCTION

Choral music is performed in a group using a superposition of melodic lines to form diverse chords and can be sung a cappella or with instrumental accompaniment^(1)^. This technique requires singers to be separated by voice type in agreement with each member’s respective vocal classifications^(1)^. In youth and adult choirs, members are generally separated into four voice types; men and women with lower voices are respectively classified as basses and contraltos, while men and women with higher voices are classified as tenors and sopranos, although subdivisions can be found within voice types^(2)^.

A choral singer must be in tune and have good pneumo-phono-articulatory coordination, vocal technique, and aural perception to execute the notes written in sheet music without vocal adjustments that could harm the vocal tract and to ensure that their voice is in unison with its vocal section and in harmony with the rest of the choir^(2,3)^. For these reasons, good orientation and vocal preparation are essential.

Trained choral singers present an elevated degree of self-perception due to their ability to perceive and adjust their voice in relation to the rest of the choir^(4)^; as a result, choral singers are evaluated in various studies precisely due to this self-perception^(5)^.

However, amateur groups can be extremely heterogeneous; studies that involved choral singers’ self-perception indicated that, as with other voice professionals, this population has a higher prevalence of vocal symptoms as well as limited vocal knowledge, thereby presenting a higher risk of developing vocal problems^(5,6)^. These studies involved singers that held in-person rehearsals.

The epicenter of an outbreak of a serious acute respiratory syndrome caused by the new coronavirus (COVID-19) was in Wuhan, China before spreading to all continents. To contain the propagation of the disease, social distancing measures had to be taken, making it impossible to participate in collective, face-to-face activities^(7)^, including musical practices and rehearsals. In-person vocal practices present a high risk for the transmission of the disease^(8,9)^ and choirs composed of the elderly, for example, are thusly composed entirely by a population with higher chances of manifesting a more serious form of the disease. For these reasons, these activities began to occur remotely.

However, classes that rely on videoconference require more work and effort by professors when compared to in-person classes, additionally bringing along a string of challenges for students^(10)^. In the area of music, a home environment isn’t always appropriate or sufficiently isolated for singing activities^(10,11)^. Moreover, videoconference platforms are designed for meetings in which only one person speaks at a time, and audio and video delays can occur even with good internet connections, not to mention audio quality issues such as dropped audio and frequency suppression^(10,11)^.

While remote activities have their benefits, they do not substitute traditional classrooms^(10)^. Regarding choral music in particular, being collective at its core, videoconferencing cannot provide choir members with proper development in all the skills necessary to sing^(12)^. Audio delay and possible problems with transmission make simultaneous singing infeasible, with some choirs instead relying on individual recordings that are then overlaid and stitched with video and audio editing programs. As such, there can be difficulties in creating a uniformed timbre among recorded voices and audio quality can vary based on recording conditions^(11)^.

A recent study^(13)^, carried out with choirs in Sweden and Norway, evaluated choral singers’ self-perception and found that these singers were affected by the COVID-19 pandemic aesthetically, emotionally, and physically.

In addition to the difficulties choral members had while learning, stemming from virtual rehearsals, the psychosocial impacts caused by social distancing are also essential to be considered. It is difficult to adapt to a lack of liberty and opportunities to see friends and relatives, as well as to the economic and professional changes provoked by the pandemic^(14,15)^. Moreover, amateur choirs are the first contact with choral singing for many singers, taking in singers who may feel more comfortable singing in a group, while individual practices and recordings can leave individuals feeling exposed^(16)^.

This study is justified by the need for knowledge about the impact that remote choir activities have had on the voice and on the routines of Brazilian choral singers since, as far as we know, no previous studies covered this topic. The objective of this study was to analyze the perception of choral singers that rehearsed virtually, including symptoms, vocal disadvantages, adaptations to singing routines, and rehearsal difficulties during social distancing as a result of the COVID-19 pandemic.

## METHODS

The present manuscript describes a quantitative, transversal study, submitted to and approved by the Research Ethics Committee of the University of Campinas (Comitê de Ética em Pesquisa da Universidade Estadual de Campinas), registered under CAAE number 39078320.9.1001.5404 and feedback number 4.577.002. One questionnaire was sent online to choral singers who continued rehearsing remotely during social isolation. Data collection was carried out between February and April of 2021.

Inclusion criteria were: be 18 years of age or older, sing in a choir since the beginning of the second semester of 2019, and have continued rehearsing remotely. Exclusion criteria were: self-reporting of health problems that could affect vocal quality and having been diagnosed with COVID-19 at any point before or during the study. Of the participants who responded to the survey, 15 reported having been diagnosed with COVID-19 and were excluded from the sample group.

A total of 141 choral singers participated in the study, coming from various amateur choral groups in the South and Southeast regions of Brazil. None had been diagnosed with COVID-19 and all had continued remote activities through April of 2021. Participants had an average age of 51 years and 11 months, with a median of 55 years; 107 (75.89%) were females and 34 (24.11%) were males.

Participants were divided into two groups based on their age range: Group 1 (G1) ranged from 18 to 54 years of age while Group 2 (G2) contained all participants aged 55 years and older. This distinction was made to divide the participants in half based on the median, which also coincides with the expected age of the end of menopause; as such, women going through this process were expected to not be present in G2.

G1 was composed of 66 singers (20 men, 30.3%; 46 women, 69.7%), aged between 18 and 54 years. The median age of the singers in this group was 40.5 years with a median time of participation in a choir of 3 years. G2 was composed of 75 singers (14 men, 18.67%; 61 women, 81.33%), aged 55 years or older. The median age of this group was 62 years and the median time of participation in a choir was 4 years.

Invitations to participate were sent by social media platforms and email. Directors and choral members were asked to forward the invitation to other colleagues and singers to increase visibility. Most choirs that participated were university-affiliated; all were amateur groups; and all had characteristics of heterogeneity regarding social class, age, and levels of vocal and musical knowledge.

The questionnaire (Supplementary Material) was designed and sent to participants using an online, free platform, covering perceptions about the voice as well as singing and rehearsing routines. It was divided into five parts: I - characterization of the participant and the choir; II - questions about COVID-19; III - questions about the voice during in-person rehearsals (before distancing); IV - questions about the voice during remote rehearsals (during distancing); and V - questions about adaptations in singing routines and possible difficulties during remote rehearsals (during distancing).

All questions were presented in a single, online questionnaire during social distancing and participants responded to the questions alone on a personal device such as their computer or cell phone, following the orientations included in the questionnaire.

The final version included 78 questions, 23 of which were asked twice, once each about the perceptions of the voice before and after distancing. For questions referring to the time before distancing, participants were instructed to respond thinking about how their voice was during in-person rehearsals. Some questions were selected from validated questionnaires that evaluate the impact of a vocal problem, perception of handicap, vocal symptoms, and fatigue, as follows, translated and culturally adapted where appropriate.

- Modern Singing Handicap Index^(17)^: questions 8 and 9;
- Classical Singing Handicap Index^(18)^: questions 2, 3, and 24;
- Voice Symptom Scale^(19)^: questions 18, 19, 20, and 21;
- Vocal Fatigue Index^(20)^: questions 22 and 23.

The final questionnaire included only questions that could respond to the objectives of the study. In addition, 12 of the questions appeared in more than one questionnaire (4-7; 10-17). Question 1, which appears in both in-person and remote rehearsal sections, was created by the researchers. Question 24 was asked only in the section about remote rehearsals (during distancing). Despite being designed for different investigations, all of the questions from validated questionnaires used a scale from 0 to 4 to describe the frequency of a given problem occurring (0 = never; 1 = almost never; 2 = sometimes; 3 = almost always; 4 = always). During the analysis, responses were compared between the pre-pandemic period and during distancing.

The other 33 questions of the questionnaire were designed by the researchers. To create a well-rounded questionnaire, the research team included a phono-audiologist and choral voice coach. Before being released to the public, a pilot test was performed for any necessary adjustments.

The average time to respond to the questionnaire was approximately 15 minutes. All of the questions had the option to refuse to answer, in case the participant did not feel comfortable responding.

The data obtained during the study underwent descriptive and inferential analyses. SPSS 25.0 software was used for statistical analyses.

The categorical qualitative variables were described by relative percentual frequency and absolute frequency. The continuous quantitative variables were described by variability measurement (standard deviation), central tendency (mean and median), and position (minimum, first quartile, third quartile, and maximum).

The inferential analysis of the comparison of discrete qualitative variables between the two groups was performed using the Wilcoxon test. The comparison of the proportions of variable categories was performed using a two-proportions z-test for equality. For the variables with more than two response categories, the category with the highest proportion was used as a reference. For inferential analyses, significance was considered using a 5% cutoff.

## RESULTS

Of the 141 choral singers that participated in the study, five (3.55%) were smokers, half (n=71; 50.35%) took medication for any chronic disease, 27 (19.15%) had any type of vocal issue, and most (n=114; 80.85%) rehearsed at least once a week.

The most commonly used platforms for rehearsals were Google Meet™ and Zoom™, together accounting for 133 (94.33%) of the participants. Of these, 42 (32.62%) exclusively used Google Meet™, 67 (47.52%) used only Zoom™, and 20 (14.18%) used both platforms. Each vocal group presented some unique characteristics.

### Group 1

Only three (4.56%) participants sang in more than one choir, two (3.03%) were smokers, 38 (57.58%) performed physical activity, and 13 (19.7%) reported some kind of vocal issue. Most of the choral members (n=46; 69.7%) rehearsed weekly, while 18 (27.28%) rehearsed two or more times per week.

Regarding the most used platforms for rehearsals in G1, 26 (39.39%) singers exclusively used Google Meet™, 25 (37.88%) exclusively used Zoom™, and ten (15.15%) used both platforms.

As for symptoms and vocal handicaps, there was a reduction in the scores of questions 12 (p=0.018), 16 (p=0.044), and 21 (p=0.005) for choral members between the ages of 18 and 54 years, as seen in Table 1.

**Table 1:** Self-perception of vocal symptoms and handicaps before and during distancing in G1

Question	Period	Average	SD	Median	p-value
1) Do you have voice problems?	BD	0.85	0.88	1.00	0.862
	DD	0.83	0.95	0.50	
2) Are you forced to limit your study/rehearsal time?	BD	0.36	0.65	0.00	0.384
	DD	0.45	0.75	0.00	
3) Do you have difficulty with dynamics like “pianissimo” or “fortissimo”?	BD	0.86	1.04	1.00	0.941
	DD	0.82	0.96	1.00	
4) Do you take medication to hide vocal problems?	BD	0.23	0.67	0.00	0.792
	DD	0.24	0.72	0.00	
5) Does your voice crack?	BD	0.80	0.93	1.00	0.525
	DD	0.71	0.82	0.50	
6) Do you get anxious about singing during rehearsal?	BD	1.08	1.13	1.00	0.498
	DD	0.98	1.12	0.50	
7) Do you get worried when you are asked to repeat a warmup or musical segment?	BD	0.77	1.05	0.00	0.084
	DD	1.02	1.12	1.00	
8) Are you unhappy with your singing voice?	BD	0.97	1.02	1.00	0.870
	DD	0.92	1.06	0.50	
9) Are you ashamed when singing?	BD	1.20	1.22	1.00	0.261
	DD	1.06	1.19	1.00	
10) Do you have problems controlling your breathing while singing?	BD	1.30	0.89	1.50	0.192
	DD	1.15	0.98	1.00	
11) Do you feel like you have a weak or airy voice?	BD	0.98	1.02	1.00	0.374
	DD	0.88	0.92	1.00	
12) Do you feel your voice can be hoarse or scratchy?	BD	0.77	0.94	0.50	0.018*
	DD	0.50	0.69	0.00	
13) Is singing a difficult or tiring task?	BD	0.62	0.86	0.00	0.674
	DD	0.58	0.77	0.00	
14) Does your voice tire easily during presentations/rehearsals/recordings?	BD	1.11	0.88	1.00	0.266
	DD	0.95	1.00	1.00	
15) Does your singing ability vary from day to day?	BD	1.20	0.98	1.00	0.063
	DD	1.38	0.96	1.00	
16) Does your throat hurt?	BD	0.79	0.87	1.00	0.044*
	DD	0.53	0.85	0.00	
17) Do you have a generally raspy or scratchy voice?	BD	0.47	0.85	0.00	0.143
	DD	0.33	0.64	0.00	
18) Do you lose your voice?	BD	0.44	0.66	0.00	0.058
	DD	0.30	0.58	0.00	
19) Do you cough or feel the need to clear your throat?	BD	1.18	1.09	1.00	0.059
	DD	0.97	1.01	1.00	
20) Do you feel like you have something stuck in your throat?	BD	0.74	1.01	0.00	0.941
	DD	0.71	1.08	0.00	
21) Do you have throat infections?	BD	0.64	0.95	0.00	0.005*
	DD	0.38	0.67	0.00	
22) Does it hurt to talk?	BD	0.61	0.99	0.00	0.490
	DD	0.55	0.91	0.00	
23) Do you feel improvements when you rest your voice?	BD	0.56	0.79	0.00	0.672
	DD	0.50	0.73	0.00	

The p-value was generated using Wilcoxon’s signed-rank test

* statistically significant variables

**Caption**: BD = before distancing; DD= during distancing; SD=standard deviation

Question 24 of the questionnaire shows that the participants in G1 did not take more time to warm up their voices when social distancing began (median 1.00; *almost never*).

When distancing began, choral members in G1 reported the absence of allergic symptoms (n=56; p<0.001), bruxism or teeth clenching (n=52; p<0.001), and gastroesophageal reflux (n=55; p=0.002) compared to before distancing. More than half (n=38; 57.58%) of the choral members in this age range reported developing anxiety during distancing; however, this did not show significance by the two proportions z-test for equality.

Lastly, Table 2 shows the proportions of respondents about the difficulties these singers reported during rehearsals.

**Table 2:** Proportions of difficulties reported by choral members during rehearsals in G1

Variable and categories	n	%	p-valor
1. Concentrating during videoconference is			
Harder	38	57.58	Ref.
Easier	8	12.12	0.022*
I don’t see a difference	19	28.79	0.044*
I prefer not to answer	1	1.52	0.005*
2. a) I get nervous when I have to sing alone during videoconference rehearsals.			
I agree.	40	60.61	Ref.
I disagree.	22	33.33	0.04*
I prefer not to answer	4	6.06	0.04*
2. b) It’s harder to sing individually than in a group.			
I agree.	45	68.18	Ref.
I disagree.	20	30.30	0.003*
I prefer not to answer	1	1.52	<0.001*
2. c) It isn’t easy to find a silent place to study or rehearse and this is a problem for my performance in online rehearsals.			
I agree.	37	56.06	Ref.
I disagree.	28	42.42	0.268
I prefer not to answer	1	1.52	<0.001*
2. d) I can’t project my voice in the place that is available for me to study.			
I agree.	32	48.48	0.746
I disagree.	34	51.52	
2. e) My singing practice time has considerably reduced during the pandemic.			
I agree.	46	69.70	0.004*
I disagree.	20	30.30	
2. f) I am ashamed to record videos of myself singing alone.			
I agree.	25	37.88	0.062
I disagree.	41	62.12	
2. g) I have a lot of difficulties when dealing with technology.			
I agree.	11	16.67	<0.001*
I disagree.	55	83.33	
2. h) My connection isn’t always good, and this is a problem for my rehearsal quality.			
I agree.	21	31.82	0.004*
I disagree.	45	68.18	
2. i) Recording the same song many times (video or audio) makes me stressed.			
I agree.	44	66.67	0.011*
I disagree.	22	33.33	
2. j) Recording the same song many times (video or audio) makes my voice tired.			
I agree.	35	53.03	0.628
I disagree.	31	46.97	
2. k) I feel that my inexperience makes it more difficult for me to sing alone.			
I agree.	23	34.85	0.042*
I disagree.	41	62.12	Ref.
I prefer not to answer	2	3.03	0.105
2. l) I get nervous when I sing alone during rehearsal and my voice doesn’t come out the way I want.			
I agree.	28	42.42	0.268
I disagree.	37	56.06	Ref.
I prefer not to answer	1	1.52	<0.001*
2. m) My breathing was better before distancing.			
I agree.	36	54.55	Ref.
I disagree.	29	43.94	0.381
I prefer not to answer	1	1.52	<0.001*

The p-value was generated with the two-sample z-test for proportions

* statistically significant variable

**Caption**: n = absolute frequency; % = percentual relative frequency; Ref = category with the highest proportion, used as comparison reference

### Grupo 2

In this group, six (7.98%) singers participated in more than one choir, three (4%) were smokers, 49 (65.33%) performed physical activities, and 14 (18.67%) reported any type of vocal issue. Most of the singers (n=51; 68%) rehearsed once per week, 4 (5.33%) rehearsed two or three times per week, and 16 (21.33%) rehearsed twice per month.

As for the platforms used during rehearsals, 42 (56%) singers rehearsed via Zoom™, 20 (26.67%) by Google Meet™, and 10 (13.33%) reported using both platforms.

Regarding vocal symptoms and handicaps, in this age group of 55 years or older and compared to the time before distancing, there were increases in the scores of questions 1 (p=0.002), 2 (p=0.009), 7 (p=0.004), 13 (p=0.022), 14 (p=0.002), 15 (p=0.007), as seen in Table 3. Under these same conditions, there were decreases in the scores to questions 16 (p=0.009) and 21 (p=0.004).

**Table 3:** Self-perception of vocal symptoms and handicaps before and during distancing in G2

Question	Period	Average	SD	Median	p-value
1) Do you have voice problems?	BD	0.76	0.77	1.00	0.002*
	DD	1.19	1.10	1.00	
2) Are you forced to limit your study/rehearsal time?	BD	0.24	0.54	0.00	0.009*
	DD	0.51	0.83	0.00	
3) Do you have difficulty with dynamics like “pianissimo” or “fortissimo”?	BD	0.60	0.79	0.00	0.400
	DD	0.68	0.95	0.00	
4) Do you take medication to hide vocal problems?	BD	0.07	0.25	0.00	0.317
	DD	0.04	0.20	0.00	
5) Does your voice crack?	BD	0.67	0.83	0.00	0.697
	DD	0.69	0.91	0.00	
6) Do you get anxious about singing during rehearsal?	BD	0.88	1.01	1.00	0.152
	DD	1.07	1.20	1.00	
7) Do you get worried when you are asked to repeat a warmup or musical segment?	BD	0.64	1.02	0.00	0.001*
	DD	1.00	1.21	1.00	
8) Are you unhappy with your singing voice?	BD	0.85	0.91	1.00	0.110
	DD	1.00	1.15	1.00	
9) Are you ashamed when singing?	BD	0.81	0.90	1.00	0.233
	DD	0.91	1.16	0.00	
10) Do you have problems controlling your breathing while singing?	BD	1.01	0.91	1.00	0.051
	DD	1.23	0.95	1.00	
11) Do you feel like you have a weak or airy voice?	BD	0.81	0.82	1.00	0.041*
	DD	1.01	1.10	1.00	
12) Do you feel your voice can be hoarse or scratchy?	BD	0.76	0.82	1.00	0.176
	DD	0.63	0.88	0.00	
13) Is singing a difficult or tiring task?	BD	0.33	0.60	0.00	0.022*
	DD	0.56	0.95	0.00	
14) Does your voice tire easily during presentations/rehearsals/recordings?	BD	0.67	0.74	1.00	0.002*
	DD	1.04	0.96	1.00	
15) Does your singing ability vary from day to day?	BD	0.83	0.88	1.00	0.007*
	DD	1.11	1.03	1.00	
16) Does your throat hurt?	BD	0.56	0.76	0.00	0.009*
	DD	0.36	0.65	0.00	
17) Do you have a generally raspy or scratchy voice?	BD	0.43	0.70	0.00	0.113
	DD	0.56	0.79	0.00	
18) Do you lose your voice?	BD	0.31	0.52	0.00	0.384
	DD	0.36	0.73	0.00	
19) Do you cough or feel the need to clear your throat?	BD	0.88	0.84	1.00	0.313
	DD	0.79	0.89	1.00	
20) Do you feel like you have something stuck in your throat?	BD	0.56	0.76	0.00	0.766
	DD	0.59	0.89	0.00	
21) Do you have throat infections?	BD	0.45	0.66	0.00	0.004*
	DD	0.25	0.55	0.00	
22) Does it hurt to talk?	BD	0.31	0.61	0.00	0.684
	DD	0.28	0.58	0.00	
23) Do you feel improvements when you rest your voice?	BD	0.40	0.68	0.00	0.545
	DD	0.44	0.76	0.00	

The p-value was generated using Wilcoxon’s signed-rank test

* statistically significant variables

**Caption**: BD = before distancing; DD = during distancing; SD = standard deviation

As was the case with G1, question 24 showed that participants from G2 did not take more time to warm up their voice during social distancing (median 1.00; *almost never*).

The majority of participants in G2 self-reported that during social distancing, they did not develop anxiety (n=51; p=0.004), allergic symptoms (n=70; p<0.001), bruxism or teeth clenching (n=57; p=0.001), or gastroesophageal reflux (n=58; p=0.001).

Difficulties that choral members aged 55 and older reported having during rehearsals are shown in Table 4.

**Table 4:** Proportions of difficulties reported by choral members during rehearsals in G2

Variable and categories	n	%	p-valor
1. Concentrating during videoconference is			
Harder	35	46.67	Ref.
Easier	11	14.67	0.065
I don’t see a difference	28	37.33	0.428
Prefer not to answer	1	1.33	0.008*
2. a) I get nervous when I have to sing alone during videoconference rehearsals.			
Agree	38	50.67	Ref.
Disagree	36	48.00	0.797
Prefer not to answer	1	1.33	<0.001*
2. b) It’s harder to sing individually than in a group.			
Agree	53	70.67	Ref.
Disagree	21	28.00	0.001*
Prefer not to answer	1	1.33	<0.001*
2. c) It isn’t easy to find a silent place to study or rehearse and this is a problem for my performance in online rehearsals.			
Agree	40	45.98	0.234
Disagree	47	54.02	Ref.
2. d) I can’t project my voice in the place that is available for me to study.			
Agree	34	39.08	0.007*
Disagree	53	60.92	Ref.
2. e) My singing practice time has considerably reduced during the pandemic.			
Agree	54	62.07	Ref.
Disagree	32	36.78	0.081
Prefer not to answer	1	1.15	<0.001*
2. f) I am ashamed to record videos of myself singing alone.			
Agree	33	37.93	0.015*
Disagree	54	62.07	Ref.
2. g) I have a lot of difficulties when dealing with technology.			
Agree	26	29.89	<0.001*
Disagree	61	70.11	Ref.
2. h) My connection isn’t always good, and this is a problem for my rehearsal quality.			
Agree	30	34.48	0.007*
Disagree	57	65.52	Ref.
2. i) Recording the same song many times (video or audio) makes me stressed.			
Agree	45	51.72	Ref.
Disagree	40	45.98	0.553
Prefer not to answer	2	2.30	0.184
2. j) Recording the same song many times (video or audio) makes my voice tired.			
Agree	46	52.87	Ref.
Disagree	40	45.98	0.309
Prefer not to answer	1	1.15	<0.001*
2. k) I feel that my inexperience makes it more difficult for me to sing alone.			
Agree	36	41.38	0.032*
Disagree	51	58.62	Ref.
2. l) I get nervous when I sing alone during rehearsal and my voice doesn’t come out the way I want.			
Agree	36	41.38	0.081
Disagree	50	57.47	Ref.
Prefer not to answer	1	1.149	<0.001*
2. m) My breathing was better before distancing.			
Agree	42	48.28	0.669
Disagree	44	50.57	Ref.
Prefer not to answer	1	1.15	<0.001*

The p-value was generated with the two-sample z-test for proportions

* statistically significant variable

**Caption**: n = absolute frequency; % = percentual relative frequency; Ref = category with the highest proportion, used as comparison reference

**Figure 1:**
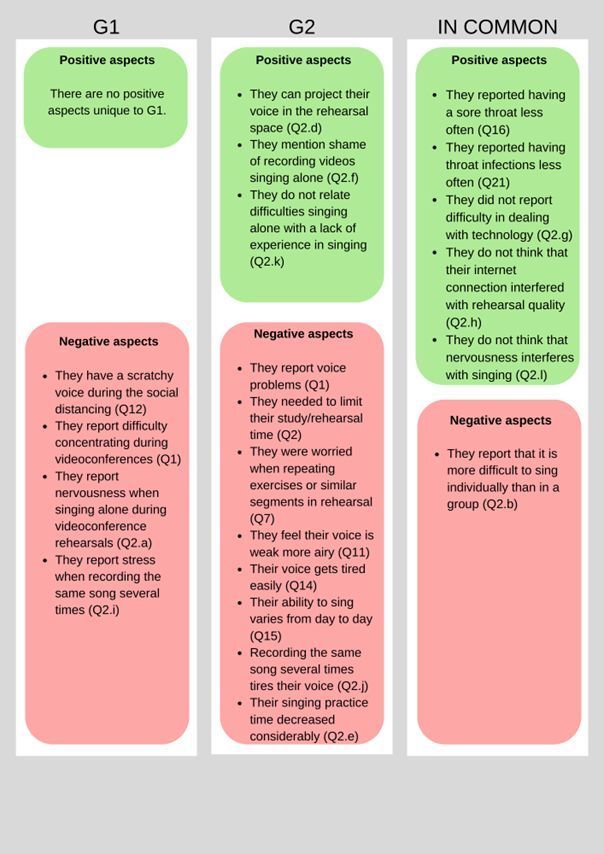
Positive and negative aspects that were reported, showing the similarities and differences between the groups

Statistically significant data from the above findings in Tables 1-4 are presented in Figure 1, showing the positive and negative aspects reported by each group, as well as those found in common between them.

## DISCUSSION

The maintenance of amateur choral groups was greatly challenged by the onset of the COVID-19 pandemic. Group singing became infeasible due to the dispersion of aerosols^(21)^ and this activity was shifted to a virtual format, placing singers and their conductors in foreign situations in respect to education and learning. Participation in amateur choirs, despite lacking the levels of responsibility of their professional counterparts, is considered to be an activity that gives great pleasure and personal satisfaction. With social distancing, the continuation of rehearsals held even greater worth to satisfy social needs.

Singers, conductors, and vocal coaches were all required to adopt a new format for musical studies, consequently altering singing and rehearsal routines. These alterations may result in the appearance of certain vocal symptoms and handicaps, as observed in this study. The results of this study show different points of view regarding these aspects in the two age groups studied.

When rehearsing online, group singing is not possible and, at times, the conductor may request a singer to enable their microphone and execute a certain musical task while everyone else listens. This type of situation can justify the worry in repeating a vocal exercise or musical phrase that was expressed by G2, as well as the nervousness about singing alone during the videoconference rehearsals, as expressed by G1. Worrying about singing alone was also reported by young singers in another study^(16)^.

The participants in both groups did not attribute the difficulty of singing alone to inexperience with singing, suggesting that nervousness plays a key role in this problem.

The responses by the participants in G1 suggest that shame or worry did not have an impact on vocal or musical quality; nonetheless, the participants in this group reported anxiety. Both anxiety and shame are known to be predictive factors for measurements of vocal acoustic intensity and a certain level of disturbance^(22)^.

As seen from the data in Tables 1 and 3, the vocal symptoms and alterations differed between groups. Specifically referring to vocal symptoms and alterations in vocal quality, some factors may be associated with the vocal issues reported by members of each group, one of which is a reduction in singing practice time, which can cause a loss in vocal conditioning in which the voice does not respond in the way a singer intends^(23)^. Moreover, age-related effects are also likely to be at play.

G1, for example, may have experienced an increase in voice use due to remote work scenarios, as shown by a previous study^(24)^, suggesting that remote work may increase the risk for developing vocal disorders, potentially justifying the worsening of vocal quality that was reported by this group. Whereas G2 likely passed through a period in which they used their voice less frequently, which might have triggered a loss in vocal resistance and consequent fatigue, vocal weakness or airiness, and variations in the voice in daily life, as reported by this group. The social interaction function of choral singing is also important to be considered^(13)^, which was restricted by online rehearsals.

Although rehearsal and vocal training routines were influenced by the changes imposed by social distancing, the groups did not relate difficulties with technology or connection with quality of learning during rehearsal. Recent literature reports that the difficulty of adapting to the use of technology during the pandemic occurred only during the first few weeks of social distancing^(25)^.

Regardless, the use of videoconferencing for online rehearsal caused discomfort, especially in G1, including difficulty in concentrating, as shown in Table 2. One hypothesis behind this finding is the general discomfort caused by online environments, such as digital fatigue caused by the obligation of being constantly online^(26)^ and cognitive fatigue that negatively affects engagement and how much choral members obtain from practicing^(27)^. Moreover, some studies^(28,29)^ have specifically discussed online activities and the discomfort students have about turning on their cameras.

During remote activities, all participants can see each other’s faces at all times, which can be exhausting; and some people may also not want to show the environment they are in during rehearsals. One possible explanation for this factor having affected mainly G1 is that the participants in this group, in addition participating in rehearsals, also likely worked remotely^(24)^.

Tables 2 and 4 also show differences in how the impact of the environment in which the rehearsals were held was perceived between the groups. The dynamics and environment that online education brought are not as favorable as traditional learning situations^(10)^. In the specific case of singing, finding a place to study is even more difficult, since not only is silence required, but there must also be at least a small amount of acoustic isolation to not bother neighbors and relatives. These factors are likely to have caused difficulties specifically for G1.

The measures adopted during COVID-19 also prevented the dissemination of other viruses and bacteria^(30)^ which can possibly explain the reduction in throat pain and infection reported by both groups.

One limitation of this study was not having included professional activity and other types of vocal activity beyond choral singing in the questionnaire.

Due to the possibility of exposure, it is important that different strategies for remote work be proposed for these populations. Participants between 18 and 54 years of age could benefit from vocal exercises and musical tasks performed outside of rehearsal times. Participants aged 55 years and older would benefit from more frequent rehearsals and a daily practice routine aimed at improving vocal resistance. Both groups would benefit from vocal orientations regarding how to best care for one’s voice during daily life.

The effects of the pandemic and the consequent social distancing are still an important matter for the vocal studies, since it deals with a situation that is still recent and not completely understood. It can be hoped that as time goes on, new discoveries will be made and with each passing day, we learn how to deal with distancing and its effects. The findings of this study contribute to Voice Studies and can aid in the organization of virtual rehearsals, in accordance with the similarities and differences seen in each of the groups that were analyzed. Nonetheless, work with choral singers should also consider the needs and the individual experience of each singer.

## CONCLUSION

When social distancing was enforced, choral singers found it hard to sing over videoconference and had reductions in vocal practice time. Choral singers did not find that connection quality was a factor that negatively affected rehearsal productivity, nor did they report difficulties with technology. Both groups reported a lower number of throat infections during this period. Choral singers between the ages of 18 and 54 years reported more emotional symptoms, while those aged 55 years and up reported more vocal symptoms.

## Supplementary Material

This article is accompanied by supplementary material.

Legend ANNEX I - QUESTIONNAIRE.

This material is available as part of the online version of the article on the following https://doi.org/10.1590/2317-1782/20232021175pt

## References

[1] Barreto CB (1973). Canto coral: organização e técnica de coro..

[2] Behlau M, Feijó D, Madazio G, Rehder MI, Azevedo R, Ferreira AE, Behlau M (2005). Voz: o livro do especialista..

[3] Vieira RH, Gadenz CD, Cassol M (2015). Longitudinal study of vocal characterization in choral singing. Rev CEFAC.

[4] Aquino FS, Teles LCS (2013). Autopercepção vocal de coristas profissionais. Rev CEFAC.

[5] Ravall S, Simberg S (2020). Voice disorders and voice knowledge in choir singers. J Voice.

[6] Rezende G, Irineu RA, Dornelas R (2015). Coro universitário: autopercepção de sintomas vocais e desvantagem vocal no canto. Rev CEFAC.

[7] Velavan TP, Meyer CG (2020). The COVID-19 epidemic. Trop Med Int Health.

[8] Charlotte N (2020). High rate of SARS-CoV-2 transmission due to choir practice in france at the beginning of the COVID-19 pandemic. J Voice.

[9] Hamner L, Dubbel P, Capron I, Ross A, Jordan A, Lee J (2020). High SARS-CoV-2 attack rate following exposure at a choir practice: Skagit County, Washington, March 2020. MMWR Morb Mortal Wkly Rep.

[10] Tragtenberg LR (2020). Live voice in remote activity: possibility and specificities. Rebento..

[11] Matos RA (2020). Possibilidades de ensino remoto de música na educação básica pautadas no Materrial Música Br. Música na Educação Básica.

[12] Egbono F, Ndigwe C (2017). Church choir online communication and music recording and streaming system. International Journal of Computer Applications Technology and Research..

[13] Theorell T, Kowalski J, Theorell AML, Horwitz EB (2020). Choir singers without rehearsals and concerts? A questionnaire study on perceived losses from restricting choral singing during the Covid-19 pandemic. J Voice.

[14] Corvo E, Caro W (2020). COVID-19 and spontaneous singing to decrease loneliness, improve cohesion, and mental well-being: an Italian experience. Psychol Trauma.

[15] Helding L, Carroll TL, Nix J, Johns MM, LeBorgne WD, Meyer D (2020). COVID-19 after effects: concerns for singers. J Voice.

[16] Hogle LA (2020). Fostering singing agency through emotional differentiation in an inclusive singing environment. Res Stud Music Educ.

[17] Moreti F, Rocha C, Borrego MCM, Behlau M (2011). Desvantagem vocal no canto: análise do protocolo Índice de Desvantagem para o Canto Moderno - IDCM. Rev Soc Bras Fonoaudiol.

[18] Ávila MEB, Oliveira G, Behlau M (2010). Índice de desvantagem vocal no canto clássico (IDCC) em cantores eruditos. Pro Fono.

[19] Moreti F, Zambon F, Oliveira G, Behlau M (2012). Equivalência cultural da versão Brasileira da Voice Symptom Scale: VoiSS. J Soc Bras Fonoaudiol.

[20] Zambon F, Moreti F, Nanjundeswaran C, Behlau M (2017). Cross-cultural adaptation of the Brazilian version of the Vocal Fatigue Index - VFI. CoDAS.

[21] Castillo-Allendes A, Contreras-Ruston F, Cantor-Cutiva LC, Codino J, Guzman M, Malebran C (2021). Voice Therapy in the context of the COVID-19 Pandemic: guidelines for clinical practice. J Voice.

[22] Monti E, Kidd DC, Carroll LM, Castano E (2017). What’s in a singer’s voice: the effect of attachment, emotions and trauma. Logoped Phoniatr Vocol.

[23] Behlau M, Moreti F, Pecoraro G (2014). Customized vocal conditioning for singing professional voice users - case report. Rev CEFAC.

[24] Siqueira LTD, Santos AP, Silva RLF, Moreira PAM, Vitor JS, Ribeiro VV (2020). Vocal self-perception of home office workers during the COVID-19 pandemic. J Voice.

[25] Velho FD, Herédia VBM (2020). Quarantined senior citizens and the impact of technology on their life. Rev Rosa Dos Ventos: Turismo e Hospitalidade..

[26] Alevizou G. (2020). Virtual schooling, Covid-gogy and digital fatigue.

[27] Hwang M, Hong J, Tai K, Chen J, Gouldthorp T (2020). The relationship between the online social anxiety, perceived information overload and fatigue, and job engagement of civil servant LINE users. Gov Inf Q.

[28] Oregon State University (2020). Zoom camera pros & cons.

[29] Trust T, Goodman L (2023). Cameras optional? Examining student camera use from a learner-centered perspective. TechTrends.

[30] Burns AAC, Gutfraind A (2021). Effectiveness of isolation policies in schools: evidence from a mathematical model of influenza and COVID-19. PeerJ.

