# Supplementary material for: Social distancing and choral singing during the Covid-19 pandemic: challenges and vocal symptoms of chorists
Source: Codas. 2023 Oct 23;35(6):e20210175. doi: 10.1590/2317-1782/20232021175en (PMC10702703; doi:10.1590/2317-1782/20232021175en)
Supplement: Legend [file codas-35-6-e20210175-Suppl.pdf]

ANEXO I – QUESTIONÁRIO

I – IDENTIFICAÇÃO

1. E-mail: ____
2. Sexo: ☐Masculino ☐Feminino ☐Não quero informar
3. Idade: ___________ ☐Não quero informar
4. Fuma? ☐Sim ☐Não ☐Recusa
5. Faz uso de algum medicamento continuamente? ☐Sim ☐Não ☐Recusa
   Se sim, qual _______________
6. Tem alguma queixa de voz? ☐Sim ☐Não ☐Recusa
7. Mantém atividade física durante o distanciamento social?
   ☐Sim ☐Não ☐Recusa
8. Desenvolveu alguma das queixas a seguir durante o distanciamento?

☐Aumento ou aparecimento de alergias

☐Problemas com o sono

☐Ansiedade

☐Bruxismo ou apertamento dentário

☐Refluxo gastroesofágico

☐Não desenvolvi nenhum sintoma

☐Recusa

9. Já teve COVID-19?

☐Sim e já me recupeirei ☐Estou com COVID-19 ☐Nunca tive COVID-19.

☐Recusa

10. Nome do coro: ____
11. Desde quando está no coro em questão? Semestre _____ Ano _____
12. Qual a frequência de ensaios não-presenciais do coro?

☐Menos de 1x por semana

☐1x por semana

☐até 3x na semana

☐mais de 3x na semana

☐Recusa

II – COVID-19

Essa parte do questionário é exibida aos participantes que já tiveram COVID-19 e a abordará os sintomas e possíveis sequelas. Caso não se sinta confortável em falar sobre o assunto, você pode pular esta etapa.

Gostaria de falar sobre os sintomas e as sequelas de COVID-19?

☐ Sim, sem problemas. ☐ Não, por favor pule esta parte.

As questões abaixo são referentes ao período que estava com COVID-19 e as sequelas da doença.

1) Quando contraiu a doença, quais sintomas apresentou?

☐Tosse ☐Febre

☐Coriza
☐Dor de garganta
☐Dificuldade para respirar
☐Perda de olfato (anosmia)
☐Alteração do paladar (ageusia)
☐Distúrbios gastrintestinais (náuseas/vômitos/diarreia)
☐Cansaço (astenia)
☐Diminuição do apetite (hiporexia)
☐Dispneia (falta de ar)
☐Não apresentei sintomas
☐ Não quero responder

2) Tem algum dos seguintes problemas de saúde

☐Baixa imunidade
☐Hipertensão
☐Diabetes
☐Doença arterial coronariana
☐Bronquite
☐Asma
☐ Doença de Parkinson
☐ Alterações isquêmicas do sistema nervo central
☐ Não quero responder

3) Ficou hospitalizado? ☐Sim ☐Não ☐Recusa

4) Foi entubado? ☐Sim ☐Não ☐Recusa

5) Com relação às sequelas da COVID-19, marque os itens que passou a manifestar:

☐Sintomas respiratórios
☐Sintomas cognitivos
☐Sintomas psicológicos
☐Cansaço/fadiga físico
☐Cansaço/fadiga vocal
☐Dor nas costas
☐Dor nas articulações
☐Falta de ar (dispineia)
☐Tosse
☐Paralisia ou paresia de prega vocal
☐Neuropatia sensorial da laringe
☐Engasgos
☐Não fiquei com nenhuma sequela
☐Outra _______________
☐Recusa

III – As questões abaixo referem-se aos ensaios presencias (ANTES do distanciamento social):

Classifique as questões abaixo:

		Nunca	Quase nunca	Às vezes	Quase sempre	Sempre	Recusa
**1.**	Antes do distanciamento social eu tinha problemas com a minha voz	☐	☐	☐	☐	☐	☐
**2.**	Por causa do meu problema de voz eu era forçado a limitar meu tempo habitual de estudo/ensaio – IDCC	☐	☐	☐	☐	☐	☐
**3.**	Tinha dificuldade de fazer dinâmicas como “pianíssimo” ou “fortíssimo” IDCC	☐	☐	☐	☐	☐	☐
**4.**	Tomava remédios continuamente para mascarar meu problema de voz IDCC	☐	☐	☐	☐	☐	☐
**5.**	Minha voz quebrava – IDCM	☐	☐	☐	☐	☐	☐
**6.**	Ficava ansioso para cantar durante um ensaio – IDCC	☐	☐	☐	☐	☐	☐
**7.**	Ficava preocupado com o problema de voz) quando me pediam para repetir um vocalize ou uma frase musical – IDCC	☐	☐	☐	☐	☐	☐
**8.**	Minha voz cantada me deixava chateado – IDCM	☐	☐	☐	☐	☐	☐
**9.**	Tinha vergonha de cantar – IDCM	☐	☐	☐	☐	☐	☐
**10.**	Tinha problemas com o controle da respiração para o canto – IDCC	☐	☐	☐	☐	☐	☐
**11.**	Sentia minha voz fraca ou tinha ar na voz – IDCC	☐	☐	☐	☐	☐	☐
**12.**	Sentia minha voz rouca – IDCC	☐	☐	☐	☐	☐	☐
**13.**	Cantar era uma tarefa difícil ou cansativa – IDCC	☐	☐	☐	☐	☐	☐
**14.**	Minha voz ficava facilmente cansada durante as apresentações ou ensaios – IDCC	☐	☐	☐	☐	☐	☐
**15.**	Minha habilidade para cantar variava dia a dia – IDCM	☐	☐	☐	☐	☐	☐

16.	Minha garganta doía – ESV	☐	☐	☐	☐	☐	☐
17.	A voz era rouca – ESV	☐	☐	☐	☐	☐	☐
18.	Perdia a voz – ESV	☐	☐	☐	☐	☐	☐
19.	Tinha tosse ou pigarro – ESV	☐	☐	☐	☐	☐	☐
20.	Sentia alguma coisa parada na garganta – ESV	☐	☐	☐	☐	☐	☐
21.	Tinha infecções de garganta – ESV	☐	☐	☐	☐	☐	☐
22.	Quando eu falava muito sentia dor para falar IFV	☐	☐	☐	☐	☐	☐
23.	Quando eu descansava, minha voz melhorava IFV	☐	☐	☐	☐	☐	☐

IV – As questões abaixo referem-se aos ensaios NÃO-presencias (DURANTE o distanciamento social):

Classifique as questões abaixo em: Nunca, Quase nunca, Às vezes, Quase sempre e Sempre:

		Nunca	Quase nunca	Às vezes	Quase sempre	Sempre	Recusa
**1.**	Após o distanciamento tenho tido problemas de voz	☐	☐	☐	☐	☐	☐
**2.**	Por causa do meu problema de voz sou forçado a limitar meu tempo habitual de estudo/ensaio - IDCC	☐	☐	☐	☐	☐	☐
**3.**	Tenho dificuldade de fazer dinâmicas como “pianíssimo” ou “fortíssimo” IDCC	☐	☐	☐	☐	☐	☐
**4.**	Tomo remédios continuamente para mascarar meu problema de voz IDCC	☐	☐	☐	☐	☐	☐
**5.**	Minha voz quebra - IDCM	☐	☐	☐	☐	☐	☐
**6.**	Fico ansioso para cantar durante um ensaio - IDCC	☐	☐	☐	☐	☐	☐
**7.**	Fico preocupado quando me pedem para repetir um vocalize ou uma frase musical - IDCC	☐	☐	☐	☐	☐	☐
**8.**	Minha voz cantada me deixa chateado - IDCM	☐	☐	☐	☐	☐	☐
**9.**	Tenho vergonha de cantar - IDCM	☐	☐	☐	☐	☐	☐
**10.**	Tenho problemas com o controle da respiração para o canto - IDCC	☐	☐	☐	☐	☐	☐
**11.**	Sinto minha voz está fraca ou tem ar na voz - IDCC	☐	☐	☐	☐	☐	☐
**12.**	Sinto minha voz rouca - IDCC	☐	☐	☐	☐	☐	☐

**13.**	Cantar é uma tarefa difícil ou cansativa - IDCC	☐	☐	☐	☐	☐	☐
**14.**	Minha voz fica facilmente cansada quando tenho que gravar vídeos ou áudios para o regente - IDCC	☐	☐	☐	☐	☐	☐
**15.**	Minha habilidade para cantar varia dia a dia – IDCM	☐	☐	☐	☐	☐	☐
**16.**	Minha garganta dói - ESV	☐	☐	☐	☐	☐	☐
**17.**	A voz é rouca - ESV	☐	☐	☐	☐	☐	☐
**18.**	Perco a voz - ESV	☐	☐	☐	☐	☐	☐
**19.**	Tenho tosse ou pigarro - ESV	☐	☐	☐	☐	☐	☐
**20.**	Sinto alguma coisa parada na garganta - ESV	☐	☐	☐	☐	☐	☐
**21.**	Tenho infecções de garganta - ESV	☐	☐	☐	☐	☐	☐
**22.**	Quando eu falo muito, sinto dor para falar - IFV	☐	☐	☐	☐	☐	☐
**23.**	Quando eu descanso minha voz melhora – IFV	☐	☐	☐	☐	☐	☐
**24.**	Tenho levado mais tempo para aquecer a minha voz - IDCC	☐	☐	☐	☐	☐	☐

Com relação aos ensaios NÃO-presenciais, responda:

1. Me concentrar por videoconferência é:
   ☐Mais fácil ☐Mais difícil ☐Não vejo diferença ☐Recusa

2. Responda se concorda ou discorda das afirmações abaixo:

		**Concordo**	**Discordo**	**Recusa**
**a.**	Fico nervoso quando tenho que cantar sozinho nos ensaios por videoconferência	☐	☐	☐
**b.**	É mais difícil cantar individualmente que em grupo	☐	☐	☐
**c.**	Não é fácil conseguir um lugar silencioso para estudar ou ensaiar e isso atrapalha meu desempenho nos ensaios não-presenciais	☐	☐	☐
**d.**	Não posso projetar a voz no local que tenho disponível para ensaiar	☐	☐	☐
**e.**	Meu tempo de prática de canto diminuiu consideravelmente no contexto de pandemia	☐	☐	☐
**f.**	Tenho vergonha de gravar vídeos cantando sozinho	☐	☐	☐
**g.**	Tenho muita dificuldade em lidar com a tecnologia	☐	☐	☐
**h.**	Minha conexão nem sempre é boa e isso atrapalha meu rendimento no ensaio	☐	☐	☐
**i.**	Gravar várias vezes a mesma música (vídeo ou áudio) me deixa estressado	☐	☐	☐

**j.**	Gravar várias vezes a mesma música (vídeo ou áudio) deixa minha voz cansada	☐	☐	☐
**k.**	Sinto que por causa da minha inexperiência tenho mais dificuldade em cantar sozinho	☐	☐	☐
**l.**	Fico nervoso e minha voz não sai direito quando tenho que cantar sozinho no ensaio	☐	☐	☐
**m.**	Minha respiração era melhor antes do distanciamento	☐	☐	☐
