# Supplementary material for: Social distancing and choral singing during the Covid-19 pandemic: challenges and vocal symptoms of chorists
Source: Codas. 2023 Oct 23;35(6):e20210175. doi: 10.1590/2317-1782/20232021175en (PMC10702703; doi:10.1590/2317-1782/20232021175en)
Supplement: Translation

# Translation: Distanciamento social e canto coral durante a pandemia do Covid-19: desafios e sintomas vocais de coristas

**DOI:** 10.1590/2317-1782/20232021175pt

**Authors:** Diego Henrique da Cruz Martinho, Ana Carolina Constantini, Elisabeth Amin, Mara Suzana Behlau

**Affiliations:** 1 Programa de Saúde Interdisciplinaridade e Reabilitação, Universidade Estadual de Campinas - UNICAMP - Campinas (SP), Brasil.; 2 Departamento de Desenvolvimento Humano, Faculdade de Ciências Médicas, Universidade Estadual de Campinas - UNICAMP - Campinas (SP), Brasil.; 3 Centro de Estudos da Voz - CEV - São Paulo (SP), Brasil.

**Keywords:** Voz, Canto, Infecções por Coronavírus, Pandemias, Educação a Distância

## Abstract

**Objetivo:**

Analisar a percepção de coristas que realizam ensaios virtuais, quanto a sintomas, desvantagens vocais, adaptações na rotina de canto e dificuldades nos ensaios durante o distanciamento social devido a pandemia do COVID-19.

**Método:**

Aplicação de questionário *online* investigando percepções sobre a voz e rotina de canto e ensaio de 141 coristas que não tiveram a doença do COVID-19 e continuaram em atividade remota até abril de 2021. Os participantes foram divididos em dois grupos de acordo com a faixa etária: G1 (18 a 54 anos) e G2 (55 anos ou mais). Os dados passaram por análise descritiva e inferencial, considerando-se nível de significância de 5%.

**Resultados:**

Durante o distanciamento, ambos os grupos referiram diminuição do tempo de canto e acharam mais difícil cantar sozinhos. Os coristas referiram menor número de infecções de garganta nesse período. Coristas do G1 perceberam voz mais rouca, relataram dificuldade para se concentrar por videoconferência, sentiam-se nervosos ao cantarem sozinhos nos ensaios virtuais e estressados gravando várias vezes a mesma música. Coristas do G2 referiram mais sintomas vocais como: voz cansada, variando ao longo do dia, voz fraca e com mais ar.

**Conclusão:**

No distanciamento social, os coristas acharam mais difícil cantar por videoconferência e tiveram o tempo de prática de canto diminuído. Coristas mais jovens referiram mais sintomas emocionais e coristas com mais idade relataram mais sintomas vocais.

## INTRODUÇÃO

O canto coral é praticado em grupo e tem como resultado a sobreposição de linhas melódicas que formam uma diversidade de acordes, os cantores podem cantar a capela ou com acompanhamento^(1)^. Essa prática exige que os cantores sejam separados por naipes, de acordo com as suas respectivas classificações vocais^(1)^. Nos coros jovens e adultos, geralmente ocorre a separação em quatro naipes, separando as vozes graves, baixos para os homens e contraltos para as mulheres, das agudas, tenores para os homens e sopranos para as mulheres, dentro dos naipes podem haver ainda subdivisões^(2)^.

Um corista precisa ser afinado, ter boa coordenação pneumofonoarticulatória, técnica vocal e boa percepção auditiva para que execute as notas escritas na partitura sem ajustes vocais que possam agredir o trato vocal e para que sua voz soe em uníssono em seu naipe e harmonize com o restante do coro^(2,3)^. Deste modo, uma boa orientação e preparação vocal são primordiais.

Coristas treinados apresentam um grau elevado de autopercepção devido a habilidade de se perceber e ajustar sua voz em relação ao coro^(4)^, fazendo com que cantores de coro sejam avaliados em diversos estudos considerando sua autopercepção^(5)^.

Entretanto, grupos amadores podem ser extremamente heterogêneos e estudos que consideram a autopercepção de coristas amadores evidenciam que, assim como outros profissionais da voz, essa população apresenta maior prevalência de sintomas vocais e têm conhecimento vocal limitado, tendo assim mais risco de desenvolver problemas vocais^(5,6)^. Tais estudos foram feitos com coristas que realizavam ensaios presenciais.

O surto de uma síndrome respiratória aguda grave causada pelo novo coronavírus (doença do coronavírus 2019 ou COVID-19) teve seu epicentro em Wuhan na China, se espalhou em todos os continentes e, para conter a propagação da doença, fizeram-se necessárias medidas de distanciamento social que impossibilitam atividades coletivas presenciais^(7)^, dentre elas os ensaios musicais. A prática vocal presencial apresenta alto risco de transmissão da doença^(8,9)^ e coros de terceira idade, por exemplo, são compostos em sua integralidade pela população com maior chance de manifestar o quadro grave da doença, portanto essas atividades passaram a ser realizadas de forma remota.

No entanto, as aulas por videoconferência são ainda mais trabalhosas para os professores que as presenciais e também trazem uma série de desafios para os alunos^(10)^. Na área musical, nem sempre o ambiente em casa é apropriado ou suficientemente isolado para as atividades de canto^(10,11)^. As plataformas utilizadas para ensaios virtuais são projetadas para reuniões faladas em que apenas uma pessoa fala por vez e podem ocorrer atrasos na transmissão do áudio e do vídeo (*delay*) mesmo que a conexão de internet esteja boa, além de problemas de qualidade sonora como o corte ou supressão de frequências^(10,11)^.

Apesar de existirem benefícios com as atividades remotas, estas não substituem as aulas presenciais^(10)^. Para o canto coral, que é essencialmente coletivo, os ensaios por videoconferência podem não suprir as necessidades dos coristas no desenvolvimento de todas as habilidades necessárias no canto^(12)^. O *delay* e possíveis problemas na transmissão impossibilitam o canto simultâneo, sendo assim alguns coros fazem a gravação individual e posterior junção das vozes por meio de programas de edição de áudio e vídeo. Contudo, pode haver dificuldades na uniformização do timbre das vozes gravadas e o volume e a qualidade do áudio variam de acordo com as condições de gravação^(11)^.

Um estudo recente^(13)^, realizado com coros da Suécia e da Noruega, avaliou a autopercepção de coristas e evidenciou que esses cantores foram afetados pela pandemia COVID-19 nos campos estético, emocional e físico.

Para além das dificuldades no aprendizado dos coristas, no que diz respeito a ensaios virtuais, também é imprescindível considerar os impactos psicossociais causados pelo distanciamento social. É difícil adaptar-se à falta de liberdade e oportunidade de se encontrar com amigos e parentes, e adaptar-se às mudanças econômicas e profissionais provocadas pela situação de pandemia^(14,15)^. Além disso, o canto coral amador é o primeiro contato com o canto de muitos cantores, pode abrigar cantores que se sentem mais confortáveis cantando em conjunto e os ensaios e vídeos individuais podem fazer com que se sintam expostos^(16)^.

Esta pesquisa justifica-se pela necessidade de se conhecer o impacto da atividade remota de coro na voz e na rotina de coristas brasileiros, visto que até onde conhecemos, não existem estudos prévios sobre essa temática. O objetivo deste estudo foi analisar a percepção de coristas que realizam ensaios virtuais, quanto a sintomas, desvantagens vocais, adaptações na rotina de canto e dificuldades nos ensaios durante o distanciamento social devido a pandemia do COVID-19.

## MÉTODO

Trata-se de estudo quantitativo, transversal, submetido e aprovado pelo Comitê de Ética em Pesquisa da Universidade Estadual de Campinas, sob CAAE nº 39078320.9.1001.5404 e parecer nº 4.577.002. Um questionário foi enviado de forma *online* a coristas que continuaram ensaiando de forma remota durante o isolamento social. A coleta de dados foi realizada no período de fevereiro a abril de 2021.

Os critérios de inclusão foram: ter 18 ou mais, cantar em um coro desde o início do segundo semestre de 2019 e continuar realizando ensaios no formato não-presencial. Os critérios de exclusão foram: o autorrelato de problemas de saúde que possam afetar a qualidade vocal e ter sido diagnosticado com COVID-19 em algum momento. Apenas 15 participantes que tiveram COVID-19 responderam à pesquisa e estes foram excluídos da amostra.

Participaram do estudo 141 coristas de diversos coros amadores das regiões sul e sudeste do Brasil, que não tiveram a doença do COVID-19 e continuaram em atividade remota até abril de 2021. Os participantes tinham idade média de 51 anos e 11 meses e mediana de 55 anos, sendo 107 (75,89%) do sexo feminino e 34 (24,11%) do sexo masculino.

Os participantes foram divididos em dois grupos de acordo com a faixa etária: Grupo 1 (G1): 18 a 54 anos; Grupo 2 (G2): 55 anos ou mais. A escolha da faixa etária de 55 anos para divisão dos grupos se deu pela mediana da idade que, inclusive, coincidiu com a idade esperada para o fim da menopausa e, deste modo, evitou-se que mulheres que estivessem passando por este período fossem incluídas no G2.

O G1 foi composto por 66 coristas (20 homens, 30,3% e 46 mulheres, 69,7%) com faixa etária de 18 a 54 anos. A mediana da idade dos coristas deste grupo foi de 40,5 anos e a mediana do tempo de participação no coro foi 3 anos. O G2 foi composto por 75 coristas (14 homens, 18,67% e 61 mulheres, 81,33%) com faixa de idade de 55 anos ou mais. A mediana da idade dos coristas deste grupo foi de 62 anos e a mediana do tempo de participação no coro foi de 4 anos.

Os convites para participação foram enviados por meio de divulgação nas redes sociais, contato por e-mail com regentes e os coristas que receberam o convite também podiam estendê-lo a outros colegas que estivessem ensaiando de forma remota, com a estratégia de bola de neve. A maioria dos coros que participou era universitário, todos são amadores e tem como característica principal a heterogeneidade de classe social, idade e conhecimentos vocais e musicais.

O questionário (Material Suplementar), elaborado e enviado aos participantes por meio de uma plataforma virtual gratuita e *online*, abordava percepções sobre a voz e rotina de canto e ensaio dos cantores e foi dividido em cinco partes: I - caracterização do participante e do coro; II - questões referentes à doença do COVID-19; III - questões sobre a voz nos ensaios presenciais (antes do distanciamento); IV - questões sobre a voz nos ensaios não-presenciais (durante o distanciamento); V - questões sobre adaptações na rotina de canto e possíveis dificuldades nos ensaios não-presenciais (durante o distanciamento).

Todas as questões foram apresentadas em um único questionário *online* durante o distanciamento social e o participante respondeu as questões sozinho em seu computador ou celular, seguindo as orientações escritas no próprio questionário.

O instrumento final possuía 78 perguntas, sendo que 23 sobre as percepções da voz foram apresentadas duas vezes, considerando dois momentos diferentes (antes e durante o distanciamento), nas questões referentes ao momento antes do distanciamento, o participante foi instruído a responder pensando em como sua voz era nos ensaios presenciais. Tais questões foram selecionadas de questionários validados de avaliação de impacto de um problema vocal, percepção de desvantagem, sintomas vocais e fadiga:

- Índice de Desvantagem Vocal para o Canto Moderno (IDCM)^(17)^: duas questões (8 e 9);
- Índice de Desvantagem Vocal para o Canto Clássico (IDCC)^(18)^: três questões (2, 3 e 24);
- Escala de Sintomas Vocais (ESV)^(19)^: quatro questões (8, 19, 20 e 21);
- Índice de Fadiga Vocal (IFV)^(20)^: duas questões (22 e 23).

Foram incluídas no questionário final apenas as questões que poderiam responder aos objetivos do estudo. Além disso, 12 questões apareciam em mais de um questionário (4, 5, 6, 7, 10, 11, 12, 13, 14, 14, 16 e 17). A questão 1, que aparece tanto na parte sobre a voz nos ensaios presenciais quanto na dos ensaios não-presenciais, foi elaborada pelos pesquisadores. A questão 24 foi apresentada apenas na parte sobre a voz nos ensaios não-presenciais (durante o distanciamento). Apesar de realizarem investigações diferentes, todas as questões dos questionários validados utilizados neste estudo tinham cinco opções de resposta de acordo com a ocorrência do problema (0 = nunca; 1 = quase nunca; 2 = às vezes; 3 = quase sempre; 4 = sempre), para a análise foram comparados os momentos pré e durante o distanciamento de cada questão.

As demais questões do questionário (33) foram elaboradas pelos pesquisadores. Para melhor adaptação da versão final do formulário, a equipe de pesquisa incluiu na elaboração uma fonoaudióloga e preparadora vocal de coros e, antes de ser amplamente divulgado, foi feita uma aplicação piloto para eventuais ajustes.

O tempo médio para responder o questionário era de cerca de 15 minutos. Todas as questões possuíam a opção “recusa” para o caso de o participante não querer ou não se sentir confortável em responder.

Os dados do estudo passaram por análise descritiva e inferencial. O *software* utilizado foi o SPSS 25.0.

A descrição das variáveis qualitativas nominais foi realizada por meio de frequência relativa percentual e de frequência absoluta. A descrição das variáveis quantitativas contínuas foi realizada por meio de medidas de variabilidade (desvio-padrão), tendência central (média e mediana) e posição (mínimo, primeiro quartil, terceiro quartil e máximo).

A análise inferencial de comparação de variáveis qualitativas ordinais entre dois grupos dependentes foi realizada com o Teste de Wilcoxon. A comparação das proporções de categorias de variáveis foi realizada com o Teste de Igualdade de Duas Proporções. Para as variáveis com mais de duas categorias de resposta, utilizou-se como referência de comparação a categoria com maior proporção. Considerou-se um nível de significância de 5% para as análises inferenciais.

## RESULTADOS

Dos 141 coristas que participaram do estudo, cinco (3,55%) eram fumantes, metade (n=71; 50,35%) tomava algum tipo de medicamento para doença crônica, 27 (19,15%) tinha algum tipo de queixa vocal e a maioria 114 (80,85%) ensaiava pelo menos uma vez na semana.

As plataformas mais utilizadas para os ensaios foram o Google Meet™ e o Zoom™, utilizadas por 133 (94,33%) dos participantes. Destes, 42 (32,62%) utilizavam apenas o Google Meet™, 67 (47,52%) apenas o Zoom™ e 20 (14,18%) utilizavam ambas as plataformas. Algumas particularidades foram apresentadas por cada grupo.

### Grupo 1

Apenas três (4,56%) participantes cantavam em mais de um coro, dois (3,03%) eram fumantes, 38 (57,58%) realizam atividades físicas e 13 (19,7%) tinham algum tipo de queixa vocal. A maioria dos coristas, 46 (69,7%), tem ensaio musical uma vez por semana e 18 (27,28%) ensaiam mais de duas vezes na semana.

Quanto as plataformas mais utilizadas para os ensaios do G1, 26 (39,39%) coristas utilizavam apenas o Google Meet™, 25 (37,88%) apenas o Zoom™ e dez (15,15%) utilizavam ambas as plataformas.

Quanto aos sintomas e desvantagens vocais, verifica-se na Tabela 1 que houve diminuição no escore da questão 12 (p=0,018), questão 16 (p=0,044) e questão 21 (p=0,005) durante o distanciamento em coristas com idade de 18 a 54 anos.

**Tabela 1:** Autopercepção de sintomas e desvantagens vocais antes e durante o distanciamento no G1

Questionamento	Momento	Média	DP	Mediana	p-valor
1) Tem problemas com voz?	AD	0,85	0,88	1,00	0,862
	DD	0,83	0,95	0,50	
2) É forçado a limitar tempo de estudo/ensaio?	AD	0,36	0,65	0,00	0,384
	DD	0,45	0,75	0,00	
3) Dificuldade com dinâmicas como “pianíssimo” ou “fortíssimo”	AD	0,86	1,04	1,00	0,941
	DD	0,82	0,96	1,00	
4) Toma remédios para mascarar problema de voz?	AD	0,23	0,67	0,00	0,792
	DD	0,24	0,72	0,00	
5) Voz quebra?	AD	0,80	0,93	1,00	0,525
	DD	0,71	0,82	0,50	
6) Fica ansioso para cantar durante o ensaio?	AD	1,08	1,13	1,00	0,498
	DD	0,98	1,12	0,50	
7) Fica preocupado quando solicitado a repetir vocalize frase musical?	AD	0,77	1,05	0,00	0,084
	DD	1,02	1,12	1,00	
8) Voz cantada deixa chateado?	AD	0,97	1,02	1,00	0,870
	DD	0,92	1,06	0,50	
9) Tem vergonha de cantar?	AD	1,20	1,22	1,00	0,261
	DD	1,06	1,19	1,00	
10) Tem problemas com o controle da respiração no canto?	AD	1,30	0,89	1,50	0,192
	DD	1,15	0,98	1,00	
11) Sente que a voz está fraca ou com ar?	AD	0,98	1,02	1,00	0,374
	DD	0,88	0,92	1,00	
12) Sente a voz rouca?	AD	0,77	0,94	0,50	0,018*
	DD	0,50	0,69	0,00	
13) Cantar é uma tarefa difícil ou cansativa?	AD	0,62	0,86	0,00	0,674
	DD	0,58	0,77	0,00	
14) Voz cansa facilmente em apresentações/ensaios/gravações?	AD	1,11	0,88	1,00	0,266
	DD	0,95	1,00	1,00	
15) Habilidade para cantar varia dia a dia?	AD	1,20	0,98	1,00	0,063
	DD	1,38	0,96	1,00	
16) A garganta dói?	AD	0,79	0,87	1,00	0,044*
	DD	0,53	0,85	0,00	
17) A voz é rouca?	AD	0,47	0,85	0,00	0,143
	DD	0,33	0,64	0,00	
18) Perde a voz?	AD	0,44	0,66	0,00	0,058
	DD	0,30	0,58	0,00	
19) Tem tosse ou pigarro?	AD	1,18	1,09	1,00	0,059
	DD	0,97	1,01	1,00	
20) Sente algo parado na garganta?	AD	0,74	1,01	0,00	0,941
	DD	0,71	1,08	0,00	
21) Tem infecções na garganta?	AD	0,64	0,95	0,00	0,005*
	DD	0,38	0,67	0,00	
22) Quando fala muito sente dor para falar?	AD	0,61	0,99	0,00	0,490
	DD	0,55	0,91	0,00	
23) Quando descansa a voz melhora?	AD	0,56	0,79	0,00	0,672
	DD	0,50	0,73	0,00	

Teste de Wilcoxon

* variáveis estatisticamente significantes

**Legenda:** AD = antes do distanciamento; DD = durante o distanciamento; DP = desvio padrão

A questão 24 do questionário evidencia que os participantes do G1 não têm levado mais tempo para aquecer a voz durante o distanciamento social (mediana 1,00; *quase nunca*).

Quanto aos problemas que apareceram com o distanciamento dos coristas de 18 a 54 anos, houve maior proporção de coristas do G1 que não relataram aparecimento de alergias (n=56; p<0,001), bruxismo ou apertamento dentário (n=52; p<0,001), e refluxo gastroesofágico (n=55; p=0,002). Apesar de não haver significância estatística no Teste de Igualdade entre Duas Proporções, mais da metade 38 (57,58%) dos coristas dessa faixa etária relataram que desenvolveram ansiedade durante o distanciamento.

Quanto às dificuldades nos ensaios, observa-se na Tabela 2 a proporção de respostas para as dificuldades dos coristas com idade entre 18 e 54 anos.

**Tabela 2:** Proporção das dificuldades nos ensaios em coristas do G1

Variável e categorias	n	%	p-valor
1. Me concentrar por videoconferência é			
Mais difícil	38	57,58	Ref.
Mais fácil	8	12,12	0,022*
Não vejo diferença	19	28,79	0,044*
Recusa	1	1,52	0,005*
2. a) Fico nervoso quando tenho que cantar sozinho nos ensaios por videoconferência			
Concordo	40	60,61	Ref.
Discordo	22	33,33	0,04*
Recusa	4	6,06	0,04*
2. b) É mais difícil cantar individualmente que em grupo			
Concordo	45	68,18	Ref.
Discordo	20	30,30	0,003*
Recusa	1	1,52	<0,001*
2. c) Não é fácil conseguir um lugar silencioso para estudar ou ensaiar e isso atrapalha meu desempenho nos ensaios não-presenciais			
Concordo	37	56,06	Ref.
Discordo	28	42,42	0,268
Recusa	1	1,52	<0,001*
2. d) Não posso projetar a voz no local que tenho disponível para ensaiar			
Concordo	32	48,48	0,746
Discordo	34	51,52	
2. e) Meu tempo de prática de canto diminuiu consideravelmente no contexto de pandemia			
Concordo	46	69,70	0,004*
Discordo	20	30,30	
2. f) Tenho vergonha de gravar vídeos cantando sozinho			
Concordo	25	37,88	0,062
Discordo	41	62,12	
2. g) Tenho muita dificuldade em lidar com a tecnologia			
Concordo	11	16,67	<0,001*
Discordo	55	83,33	
2. h) Minha conexão nem sempre é boa e isso atrapalha meu rendimento no ensaio			
Concordo	21	31,82	0,004*
Discordo	45	68,18	
2. i) Gravar várias vezes a mesma música (vídeo ou áudio) me deixa estressado			
Concordo	44	66,67	0,011*
Discordo	22	33,33	
2. j) Gravar várias vezes a mesma música (vídeo ou áudio) deixa minha voz cansada			
Concordo	35	53,03	0,628
Discordo	31	46,97	
2. k) Sinto que por causa da minha inexperiência tenho mais dificuldade em cantar sozinho			
Concordo	23	34,85	0,042*
Discordo	41	62,12	Ref.
Recusa	2	3,03	0,105
2. l) Fico nervoso e minha voz não sai direito quando tenho que cantar sozinho no ensaio			
Concordo	28	42,42	0,268
Discordo	37	56,06	Ref.
Recusa	1	1,52	<0,001*
2. m) Minha respiração era melhor antes do distanciamento			
Concordo	36	54,55	Ref.
Discordo	29	43,94	0,381
Recusa	1	1,52	<0,001*

Teste de Igualdade de Duas Proporções

* variáveis estatisticamente significantes

**Legenda:** n = frequência absoluta; % = frequência relativa percentual; Ref = categoria de maior proporção utilizada como referência para comparação

### Grupo 2

Neste grupo, seis (7,98%) coristas cantavam em mais de um coro, três (4%) eram fumantes, 49 (65,33%) dos participantes realizam atividades físicas e 14 (18,67%) tinham algum tipo de queixa vocal. A maioria dos coristas, 51 (68%), tem ensaio musical uma vez por semana e 4 (5,33%) ensaiam até três vezes na semana e 16 (21,33%) tem ensaios quinzenais.

Quantos às plataformas utilizadas nos ensaios, 42 (56%) coristas ensaiavam pelo Zoom™, 20 (26,67%) pelo Google Meet™, e 10 (13,33%) utilizavam ambas as plataformas.

Quanto aos sintomas e desvantagens vocais, na Tabela 3 é possível observar que houve aumento no escore da questão 1 (p=0,002), questão 2 (p=0,009), questão 7 (p=0,004), questão 13 (p=0,022), questão 14 (p=0,002) e questão 15 (p=0,007) durante o isolamento, e redução no escore da questão 16 (p=0,009) e questão 21 (p=0,004), durante o distanciamento em coristas com idade superior a 55 anos.

**Tabela 3:** Autopercepção de sintomas e desvantagens vocais antes e durante o distanciamento no G2

Questionamento	Momento	Média	DP	Mediana	p-valor
1) Tem problemas com voz?	AD	0,76	0,77	1,00	0,002*
	DD	1,19	1,10	1,00	
2) É forçado a limitar tempo de estudo/ensaio?	AD	0,24	0,54	0,00	0,009*
	DD	0,51	0,83	0,00	
3) Dificuldade com dinâmicas como “pianíssimo” ou “fortíssimo”	AD	0,60	0,79	0,00	0,400
	DD	0,68	0,95	0,00	
4) Toma remédios para mascarar problema de voz?	AD	0,07	0,25	0,00	0,317
	DD	0,04	0,20	0,00	
5) Voz quebra?	AD	0,67	0,83	0,00	0,697
	DD	0,69	0,91	0,00	
6) Fica ansioso para cantar durante o ensaio?	AD	0,88	1,01	1,00	0,152
	DD	1,07	1,20	1,00	
7) Fica preocupado quando solicitado a repetir vocalize frase musical?	AD	0,64	1,02	0,00	0,001*
	DD	1,00	1,21	1,00	
8) Voz cantada deixa chateado?	AD	0,85	0,91	1,00	0,110
	DD	1,00	1,15	1,00	
9) Tem vergonha de cantar?	AD	0,81	0,90	1,00	0,233
	DD	0,91	1,16	0,00	
10) Tem problemas com o controle da respiração no canto?	AD	1,01	0,91	1,00	0,051
	DD	1,23	0,95	1,00	
11) Sente que a voz está fraca ou com ar?	AD	0,81	0,82	1,00	0,041*
	DD	1,01	1,10	1,00	
12) Sente a voz rouca?	AD	0,76	0,82	1,00	0,176
	DD	0,63	0,88	0,00	
13) Cantar é uma tarefa difícil ou cansativa?	AD	0,33	0,60	0,00	0,022*
	DD	0,56	0,95	0,00	
14) Voz cansa facilmente em apresentações/ensaios/gravações?	AD	0,67	0,74	1,00	0,002*
	DD	1,04	0,96	1,00	
15) Habilidade para cantar varia dia a dia?	AD	0,83	0,88	1,00	0,007*
	DD	1,11	1,03	1,00	
16) A garganta dói?	AD	0,56	0,76	0,00	0,009*
	DD	0,36	0,65	0,00	
17) A voz é rouca?	AD	0,43	0,70	0,00	0,113
	DD	0,56	0,79	0,00	
18) Perde a voz?	AD	0,31	0,52	0,00	0,384
	DD	0,36	0,73	0,00	
19) Tem tosse ou pigarro?	AD	0,88	0,84	1,00	0,313
	DD	0,79	0,89	1,00	
20) Sente algo parado na garganta?	AD	0,56	0,76	0,00	0,766
	DD	0,59	0,89	0,00	
21) Tem infecções na garganta?	AD	0,45	0,66	0,00	0,004*
	DD	0,25	0,55	0,00	
22) Quando fala muito sente dor para falar?	AD	0,31	0,61	0,00	0,684
	DD	0,28	0,58	0,00	
23) Quando descansa a voz melhora?	AD	0,40	0,68	0,00	0,545
	DD	0,44	0,76	0,00	

Teste de Wilcoxon

* variáveis estatisticamente significantes

**Legenda**: AD = antes do distanciamento; DD = durante o distanciamento; DP = desvio padrão

Assim como no G1, a questão 24 do questionário também evidenciou que os participantes do G2 não têm levado mais tempo para aquecer a voz durante o distanciamento social (mediana 1,00; *quase nunca*).

Sobre os problemas autorrelatados que apareceram no distanciamento em coristas com idade superior a 55 anos, a maioria relatou não ter desenvolvido ansiedade (n=51; p=0,004), alergias (n=70; p<0,001), bruxismo ou apertamento dentário (n=57; p=0,001) e refluxo gastroesofágico (n=58; p=0,001).

Quanto às dificuldades nos ensaios, a Tabela 4 mostra a proporção de respostas para as dificuldades dos coristas com a faixa de idade de 55 anos ou mais.

**Tabela 4:** Proporção das dificuldades nos ensaios em coristas do Grupo 2

Variável e categorias	n	%	p-valor
1. Me concentrar por videoconferência é			
Mais difícil	35	46,67	Ref.
Mais fácil	11	14,67	0,065
Não vejo diferença	28	37,33	0,428
Recusa	1	1,33	0,008*
2. a) Fico nervoso quando tenho que cantar sozinho nos ensaios por videoconferência			
Concordo	38	50,67	Ref.
Discordo	36	48,00	0,797
Recusa	1	1,33	<0,001*
2. b) É mais difícil cantar individualmente que em grupo			
Concordo	53	70,67	Ref.
Discordo	21	28,00	0,001*
Recusa	1	1,33	<0,001*
2. c) Não é fácil conseguir um lugar silencioso para estudar ou ensaiar e isso atrapalha meu desempenho nos ensaios não-presenciais			
Concordo	40	45,98	0,234
Discordo	47	54,02	
2. d) Não posso projetar a voz no local que tenho disponível para ensaiar			
Concordo	34	39,08	0,007*
Discordo	53	60,92	
2. e) Meu tempo de prática de canto diminuiu consideravelmente no contexto de pandemia			
Concordo	54	62,07	Ref.
Discordo	32	36,78	0,081
Recusa	1	1,15	<0,001*
2. f) Tenho vergonha de gravar vídeos cantando sozinho			
Concordo	33	37,93	0,015*
Discordo	54	62,07	
2. g) Tenho muita dificuldade em lidar com a tecnologia			
Concordo	26	29,89	<0,001*
Discordo	61	70,11	
2. h) Minha conexão nem sempre é boa e isso atrapalha meu rendimento no ensaio			
Concordo	30	34,48	0,007*
Discordo	57	65,52	
2. i) Gravar várias vezes a mesma música (vídeo ou áudio) me deixa estressado			
Concordo	45	51,72	Ref.
Discordo	40	45,98	0,553
Recusa	2	2,30	0,184
2. j) Gravar várias vezes a mesma música (vídeo ou áudio) deixa minha voz cansada			
Concordo	46	52,87	Ref.
Discordo	40	45,98	0,309
Recusa	1	1,15	<0,001*
2. k) Sinto que por causa da minha inexperiência tenho mais dificuldade em cantar sozinho			
Concordo	36	41,38	0,032*
Discordo	51	58,62	
2. l) Fico nervoso e minha voz não sai direito quando tenho que cantar sozinho no ensaio			
Concordo	36	41,38	0,081
Discordo	50	57,47	Ref.
Recusa	1	1,149	<0,001*
2. m) Minha respiração era melhor antes do distanciamento			
Concordo	42	48,28	0,669
Discordo	44	50,57	Ref.
Recusa	1	1,15	<0,001*

Teste de Igualdade de Duas Proporções

* variáveis estatisticamente significantes

**Legenda:** n = frequência absoluta; %=frequência relativa percentual; Ref = categoria de maior proporção utilizada como referência para comparação

O Figura 1, abaixo, faz uma síntese dos achados estatisticamente significantes evidenciando os aspectos positivos e negativos relatados por cada grupo e em comum entre eles, com base nas tabelas de 1 a 4.

**Figura 1:** Aspectos positivos e negativos relatados, evidenciando as semelhanças e diferenças dos grupos

## DISCUSSÃO

A pandemia do COVID-19 gerou uma série de desafios para a manutenção de atividades do canto coral amador. O canto em grupo se tornou impraticável pela dispersão de aerossóis^(21)^ e essa atividade passou a ser realizada em plataformas virtuais, o que colocou cantores e regentes frente a uma situação até então desconhecida para o ensino e a aprendizagem. Coros amadores, embora não tenham a responsabilidade dos coros profissionais, são vistos como atividade de grande prazer e satisfação pessoal. Com o distanciamento social, a possibilidade de manter os ensaios teve um valor ainda maior para satisfazer as necessidades sociais.

Coristas, regentes e preparadores vocais foram obrigados a adotar uma nova modalidade para os estudos musicais, tendo como consequência alterações na rotina de canto e ensaio que podem resultar no aparecimento de alguns sintomas e desvantagens vocais conforme observado neste estudo. Os resultados deste estudo apontaram percepções diferentes quanto a esses aspectos nas duas faixas etárias estudadas.

No ambiente *online*, o canto em grupo é impossibilitado e, por vezes, o regente solicita que algum corista abra o microfone e execute alguma tarefa musical com todos ouvindo. Esse tipo de situação pode justificar a preocupação de repetir um vocalize ou frase musical relatada pelo G2, além do nervosismo ao cantar sozinho nos ensaios por videoconferência, relatada pelo G1. A preocupação de cantar sozinho também foi relatada por cantores jovens em outro estudo^(16)^.

Os participantes de ambos os grupos não atribuíram a dificuldade de cantarem sozinhos à inexperiência com o canto, sugerindo o nervosismo como um ponto-chave desse problema.

As respostas dos participantes do G1 sugerem que a vergonha ou a preocupação não teve impacto na qualidade vocal ou musical, entretanto, os participantes deste grupo relataram ansiedade e sabe-se que ansiedade e a vergonha podem predizer medidas vocais acústicas de intensidade e certo ponto de perturbação^(22)^.

Como foi evidenciado nas Tabelas 1 e 3, os sintomas e alterações vocais diferiram entre os grupos. Quanto aos sintomas vocais e alterações na qualidade, alguns fatores podem ser associados às queixas vocais relatadas pelos grupos, um deles é a diminuição da prática de canto que pode ocasionar perda do condicionamento vocal fazendo com que a voz não responda da mesma forma às demandas^(23)^. Além disso, deve-se levar em conta prováveis particularidades de cada faixa etária.

O G1, por exemplo, pode ter tido um aumento do uso vocal no trabalho remoto como evidenciado em estudo anterior^(24)^ que sugeriu que o trabalho remoto aumenta o risco de desenvolver distúrbios vocais, isso pode justificar a piora da qualidade vocal relatada por este grupo. O G2, provavelmente, vivenciou uma diminuição do uso social da voz, o que pode ter desencadeado a perda da resistência vocal e as consequentes fadiga, voz fraca ou com ar e variações da voz no dia a dia relatadas por este grupo. É importante também considerar a função de interação social do canto coral^(13)^ que foi restringida pelos ensaios *online*.

Apesar de ser fato que a rotina dos ensaios e treinamentos de canto foram influenciados por mudanças impostas pelo distanciamento social, os grupos não relacionaram dificuldades com a tecnologia ou conexão com o rendimento no ensaio. Literatura recente relata que a dificuldade de adaptação ao uso da tecnologia na pandemia se deu apenas nas primeiras semanas do distanciamento social^(25)^.

Apesar disso, o uso da videoconferência para o ensaio online gerou desconforto especialmente no G1 como a dificuldade de concentração evidenciada na Tabela 2. Uma hipótese para esse achado pode ser o desconforto geral causado pelo ambiente online como a fadiga digital causada pela obrigação de estar o tempo todo online^(26)^ e a fadiga cognitiva que prejudica o engajamento e o rendimento dos coristas^(27)^. Além disso, alguns estudos^(28,29)^ discutem sobre as atividades online e o desconforto que os alunos têm ao ligar a câmera.

Durante a atividade remota todos os participantes podem ver os rostos uns dos outros o tempo todo, o que pode ser exaustivo, além de poderem não querer mostrar o ambiente em que se encontram durante o ensaio. Uma possível explicação para essa questão ter afetado principalmente o G1 é que os participantes deste grupo, além dos ensaios, muito provavelmente também realizem atividades de estudo e trabalho de forma remota^(24)^.

As Tabelas 2 e 4 também evidenciam diferenças na percepção do impacto do ambiente nos ensaios ocorreram entre os grupos. Sabe-se que as mudanças na dinâmica e no ambiente não são tão favoráveis quanto no ensino presencial^(10)^. No caso do canto, encontrar um lugar para estudo é ainda mais difícil, pois além de silencioso este precisa ter um mínimo de isolamento acústico para que não atrapalhe a rotina de vizinhos e familiares. Possivelmente esses fatores atrapalharam especificamente o G1.

As medidas adotadas contra o COVID-19 previnem também a disseminação de outros vírus e bactérias^(30)^ e podem explicar a diminuição das dores e infecções de garganta relatadas por ambos os grupos.

Uma limitação deste estudo foi não ter pesquisado a atividade profissional e outros tipos de atividade vocal dos participantes além do canto coral.

Diante do exposto, é importante que sejam propostas estratégias diferentes para o trabalho remoto com essas populações. Os participantes de 18 a 54 anos poderiam se beneficiar de vocalizes e tarefas musicais que não fossem executadas de forma síncrona e os com 55 anos ou mais se beneficiariam de ensaios mais frequentes e uma rotina de prática diária que tivesse como foco melhorar a resistência vocal. Ambos os grupos seriam beneficiados com orientações vocais para o cuidado com a voz no dia a dia.

Os efeitos da pandemia e do consequente distanciamento social ainda são uma grande questão para a área da voz, pois trata-se de uma situação ainda recente e não totalmente conhecida. Espera-se que com o passar do tempo novas descobertas sejam feitas e a cada dia se aprenda a lidar com o distanciamento e seus efeitos. Os achados deste estudo contribuem para a área da Voz e podem auxiliar na organização de ensaios virtuais, de acordo com as semelhanças e diferenças evidenciadas em cada grupo analisado. No entanto, o trabalho com coristas também deve considerar as necessidades e a experiência individual de cada cantor.

## CONCLUSÃO

Na vigência do distanciamento social, os coristas acharam mais difícil cantar por videoconferência e tiveram o tempo de prática de canto diminuído. Os coristas não consideraram a qualidade da conexão como fator que atrapalhasse o rendimento nos ensaios e não relataram ter dificuldade com a tecnologia. Além disso, ambos os grupos referiram menor número de infecções de garganta nesse período. Coristas com faixa de idade de 18 a 54 anos relataram mais sintomas emocionais e coristas com faixa de idade de 55 anos ou mais relataram mais sintomas vocais.

## Material Suplementar

Este artigo acompanha material suplementar.

Legenda ANEXO I - QUESTIONÁRIO.

Este material está disponível como parte da versão online do artigo na página: https://doi.org/10.1590/2317-1782/20232021175pt
